# Pseudophosphatase STYX is induced by *Helicobacter pylori* and promotes gastric cancer progression by inhibiting FBXO31 function

**DOI:** 10.1038/s41419-022-04696-x

**Published:** 2022-03-25

**Authors:** Jiansong Liu, Yichen Zang, Cunying Ma, Dandan Wang, Zhuangfei Tian, Xia Xu, Wenjuan Li, Jihui Jia, Zhifang Liu

**Affiliations:** 1 Department of Biochemistry and Molecular Biology, Key Laboratory for Experimental Teratology of Chinese Ministry of Education, School of Basic Medical Sciences, Cheeloo College of Medicine, Shandong University, 250012 Jinan, People’s Republic of China; 2 Department of Microbiology, Key Laboratory for Experimental Teratology of Chinese Ministry of Education, School of Basic Medical sciences, Cheeloo College of Medicine, Shandong University, Jinan, P. R. China

**Keywords:** Ubiquitylation, Gastric cancer

## Abstract

Gastric cancer (GC) is one of the most common malignancies in the world and ranks third in terms of cancer-related deaths. The catalytically inactive pseudophosphatase STYX (serine/threonine/tyrosine interacting protein) is a member of the protein tyrosine phosphatase family. It has been recently reported that STYX functions as a potential oncogene in different types of cancers. However, the potential role and regulatory mechanism of STYX in GC remains unknown. In this study, we find that STYX is highly expressed in GC tissues compared with adjacent noncancerous tissues and closely correlates with the prognosis of GC patients. STYX overexpression facilitates the proliferation and migration in GC cells, whereas STYX knockdown has the opposite effects. Nude mice experiments indicate that STYX knockdown in GC cells dramatically suppresses the tumor growth and lung metastasis in vivo. Mechanically, our results suggest that STYX interacts with the F-box protein FBXO31 and disrupts the degradation function of FBXO31 to its target proteins CyclinD1 and Snail1, thereby increasing the level of CyclinD1 and Snail1 in GC. STYX-mediated biological changes can be reversed by the co-expression of STYX and FBXO31 in GC cells. In addition, transcription factor c-Jun can enhance the expression of STYX in GC. The expression of STYX can also be induced by *Helicobacter pylori (H. pylori)* infection in c-Jun-dependent manner. Together, our present study suggests that STYX plays an oncogenic role in GC by inhibiting FBXO31 function and represents a potential therapeutic target and prognostic biomarker in GC.

## Introduction

Gastric cancer (GC) is one of the most common malignances in the world [1]. Due to the difficulties to diagnose at an early stage, the mortality of GC is high, ranking third in cancer-related deaths [2, 3]. Therefore, it is important to explore the molecular mechanism of GC occurrence and development and find the effective early diagnostic biomarkers and therapeutic targets for the tumor.

The catalytically inactive pseudophosphatase STYX belongs to the protein tyrosine phosphatase (PTPs) family [4]. Within the phosphatase domain of STYX, the cysteine residue at position 120 is replaced by glycine residue, resulting in its catalytic inactivity of the dephosphorylation function [5]. Despite the lack of catalytic function, the pseudophosphatase STYX has been implicated in various biological behaviors, such as spermiogenesis [6], neurite outgrowth [7], and cancer [8]. STYX exerts its function by binding to other proteins to affect the activity and function of the proteins. For example, the report from Wishart et al. showed that STYX interacts with a testicular RNA binding protein Crhsp-24 and is essential for normal spermiogenesis [6]. Reiterer et al. showed that the STYX binds to extracellular signal regulated kinases ERK1/2 to regulate their nuclear export and biological activities [9]. Another study from Reiterer et al. indicated that STYX inhibits the apoptosis of breast cancer cells by binding the F-box and WD repeat domain-containing 7 (FBXW7) protein and inhibiting the formation of SCF^FBXW7^ complex, thereby affecting the cellular levels of FBXW7 substrates [8]. Consistent with Reiterer’s report, He et al. suggested that STYX promotes the tumor growth and metastasis of colorectal cancer by inhibiting FBXW7 function [10]. Liu et al. also showed that STYX/FBXW7 axis participates in the development of endometrial cancer [11]. It has been well-known that the tumor suppressor FBXW7 belongs to the F-box protein (FBP) family and is a component of SCF (SKP1/CUL1/F-box) E3 ubiquitin ligase [12, 13]. There are three types of FBPs: FBXWs (contain a WD40 domain), FBXLs (contain leucine-rich repeats), and FBXOs (contain diverse types of domains) [13]. All FBPs contain a conserved F-box domain, which is the site to bind to SKP1 [14]. FBPs family members function as an alternative component of SCF, which is responsible for binding a variety of targets through its exchangeable substrate recognition domain near the carboxy terminus, and allows one core scaffold to mediate the ubiquitination of multiple substrates in different physiological states accurately [15, 16]. Our previous studies indicated that FBPs family member FBXO31 plays an important role in the occurrence and development of GC [17, 18]. We found that FBXO31 inhibits GC EMT by targeting Snail1 for proteasomal degradation [18]. The reports from Santra et al. showed that FBXO31 mediates cyclinD1 for proteasomal degradation to induce G1 arrest [19].

In this study, we investigated whether STYX can affect the function of FBXO31 in GC. We also determined its expression, role and regulatory mechanism in GC. Our results suggested that STYX bound to the F-box of FBXO31, thereby inhibiting the binding and degradation of FBXO31 to its target protein CyclinD1 and Snail1. Besides, we indicated that STYX was upregulated in GC tissues and promoted GC proliferation and metastasis both in vitro and in vivo. Furthermore, we confirmed that *H.pylori* infection contributed to the high expression of STYX in GC. To the best of our knowledge, this is the first report about the expression, role and regulatory mechanism of STYX in GC. Our findings indicate that STYX may act as a promising diagnostic and prognostic marker for GC patients.

## Materials and method

### Cell lines and clinical tissue samples

Human GC cell lines BGC-823, SGC-7901, HGC-27, AGS and 293T were purchased from Cell Resource Center, Institute of Biochemistry and Cell Biology at the Chinese Academy of Sciences, Shanghai, PR China. BGC-823, SGC-7901, HGC-27 and 293T cells were cultured in RPMI-1640 medium supplemented with 10% FBS. AGS cells were cultured in F12 medium with 10% FBS. All of the cells were cultured at 37 °C with 5% CO_2_.

A total of 25 paired human GC and adjacent noncancerous tissues were obtained from primary GC patients at the Central Hospital of Taian City in Shandong province in 2016. All specimens were stored at liquid nitrogen until use. The study protocol was approved by the patients and ethics committee of School of Basic Medicine, Shandong University. Informed consent was obtained from all individuals.

### siRNAs and plasmids

The chemically modified Stealth siRNAs targeting STYX,c-Jun and control siRNA were ordered from Genepharma (Shanghai, China). The sequences for STYX siRNA1, STYX siRNA2, c-Jun siRNA, and negative control siRNA were listed in Table S1. pCMV-myc-FBXO31 vector and the F-box domain deletion mutation vector (pCMV-myc-FBXO31ΔF) were kindly provided by Professor David F. Callen (Dame Roma Mitchell Cancer Research Labora-tories, Department of Medicine, University of Adelaide and Han-son Institute). STYX eukaryotic expression vector was constructed by inserting the CDS sequence of STYX into p-3×Flag-CMV. The primer sequences were listed in Table S1. The empty p-3×Flag-CMV plasmid was kindly provided by researcher Lidong Zhu (Center of Pathology, School of Medicine, Shanghai Jiao Tong University, China). c-Jun overexpression vector was purchased from Genepharma (Shanghai, China). X-tremeGENE HP Transfection Reagent (Roche Applied Science, Germany) or Hieff Trans™ Liposomal Transfection Reagent (Shanghai Yeasen BioTechnologies co Co., Ltd.) was used to transfect the plasmid into GC cells. Lipofectamine 2000 (Invitrogen, USA) was used to transfect the siRNA into GC cells. The experiment was performed according to the manufacturer’s instructions.

### Lentivirus infection

pLKO-Luciferase lentivirus harboring STYX shRNA (LV-STYX-shRNA-Puromycin) or negative control were purchased from Genechem Co. Ltd. (Shanghai, China). The lentiviruses infected GC cells in presence of polybrene. Then, the cells were selected in medium containing 2 μg/mL puromycin (Sigma, USA).

### RNA extraction, reverse transcription, and qRT-PCR

We used Trizol reagent (Invitrogen) to extract the total RNA from the GC cells or gastric tissues according to the manufacturer’s protocol. The qPCR primers for STYX and β_2_-M (β-microglobulin) were synthesized from BioSune (Shanghai, China). The primers sequences were listed in Table S1. The first-strand cDNA was synthesized with random primers. The mRNA expression level was determined with qRT-PCR using Bio-Rad cfx96tm real-time PCR system and SYBR Green kit (Vazyme Biotech Co, Ltd. NanJing, China) according to the protocol of manufacturer. Calculation of target mRNA levels was based on the CT method and normalization to human β_2_-M expression. All reactions were run in triplicate.

### Western blot analysis

Total proteins from the GC cells or gastric tissues were extracted with RIPA lysis buffer supplemented with protease inhibitors. Protein concentration was measured with the BCA Kit. The proteins were separated by SDS-PAGE and transferred to a PVDF membrane (Millipore). After blocking with TBST containing 5% nonfat milk for 2 h, the membrane was incubated with specific primary antibodies against STYX (1:1000; Abcam, ab205200), FBXO31 (1:1000; Abcam, ab86137), Snail1 (1:1000; Cell Signaling Technology, C15D3), CyclinD1 (1:1000;proteintech, 2G3G5), Myc (1:1000; TransGen Biotech, HT101E), Flag (1:1000; Sigma–Aldrich, F1804), β-actin (1:4000; Cell Signaling Technology, 8H10D10), c-Jun (1:1000; Cell Signaling Technology, 60A8), CagA (1:1000; Stanta Cruz, sc-17450) at 4 °C overnight. Then the membranes were washed in Tris-buffered saline with Tween, incubated with anti-mouse or anti-rabbit horseradish peroxidase conjugated secondary antibody for 1 h at room temperature, and developed with the enhanced chemiluminescence method (Millipore, USA). β-actin served as a loading control. CHX (cycloheximide) was from Sigma–Aldrich (5087390001).

### Co-immunoprecipitation assay

For exogenous Co-IP assay, the GC cells were seeded on a 10 cm culture plate and co-transfected with different expression vectors. After 48 h, the cells were lysed with IP lysis buffer. About 1 mg of total protein was incubated with 5 µg of antibody against Flag (Sigma–Aldrich, F1804) or Myc-tag (TransGen Biotech, HT101e) or IgG overnight at 4 °C on a vertical roller. For endogenous IP assay, the 293 T cells were inoculated on a 10 cm culture plate and lysed with IP lysis buffer. The total protein was incubated with 5ug of antibody against FBXO31 or STYX or IgG. Then the mixture was incubated with 30 µL Protein A/G PLUS Agarose beads for 4 h. The immunoprecipitated proteins were identified by western blot.

### Transwell migration assay

The GC cells with different transfection were harvested and resuspended in serum-free RPMI-1640 medium. Cells (5 × 10^4^) were seeded into the upper of 24-well chambers. RPMI-1640 medium containing 20% FBS was added to the lower chambers as a chemoattractant. After 24 h, cells on the upper surface of polycarbonate membrane were removed with cotton swabs and cells that had migrated the membrane filter were fixed with methanol and stained with crystal violet and photographed under a microscope. The number of migration cells was counted. The experiments were repeated three times in triplicate.

### Cell proliferation assays

The EdU assay was performed according to the protocol of the Cell-Light™ EdU Apollo®567 In Vitro Imaging Kit (RiboBio, Guangzhou, China). Briefly, the treated cells were seeded in 96-well plates and incubated with 50 μM EdU for 2 h at 37 °C. After being fixed with 4% paraformaldehyde, the cells were exposed to 100 μL of 1 × Apollo® reaction cocktail and then incubated with 5 μg/mL Hoechst 33342 to stain cell nuclei. Images were captured using a fluorescence microscope (Olympus, Tokyo, Japan). The percentage of EdU-positive cells was defined as the proliferation rate. The experiments were repeated three times in in sextuplicate. For the CCK-8 assay, the treated cells were seeded in 96-well plates and incubated with 100 μL 10% CCK-8 solution for 4 h at 37 °C. The absorbance was measured at 450 nm and 650 nm as reference with an Infinite M200 spectrophotometer (Tecan). The experiments were repeated three times in triplicate.

### Animal experiment

Five-week-old male BALB/c nude mice were bought from the Nanjing Biomedical Research Institute of Nanjing University (Nanjing, China). All mice were randomly assigned to the subcutaneous injection group (*n* = 6) and tail vein injection group based on “complete randomization” rules (*n* = 16). For the nude mice tumor formation experiment, BGC-823 cells (6 × 10^5^) with STYX overexpression or knockdown were harvested and resuspended in 100 μL PBS. Then the cells were injected subcutaneously into either side of the back of each nude mice with empty vector as control. After 3 days, the subcutaneous tumor growth was recorded every 2–3 days by measuring the width (W) and length (L) with vernier caliper, and the tumor volume (V) was calculated using the formula V = (W^2^ × L)/2. Three weeks after injection, the mice were euthanized, and the tumors were removed and weighed. About half of the tumor were fixed, embedded and stained with hematoxylin-eosin staining. Protein was extracted from the rest of the tumor for western blot. To investigate the effect of STYX on the metastatic ability of GC cells in nude mice, we injected BGC-823 (6 × 10^5^) cells with stable STYX knockdown into the tail veins of nude mice. The metastasis of the cells in nude mice was monitored with a small-animal in vivo imaging system. After 5 weeks, the mice were sacrificed. The lungs of the nude mice were collected, weighed, and stained with hematoxylin-eosin staining. All mice experiments were approved by the ethics committee of the School of Basic Medical Sciences, Shandong University and were performed in accordance with the guidance of animal experiments in the Laboratory Animal Center of Shandong University.

### *H.pylori* cultures and infection assay

The standard *H. pylori* strain 26695 and SS1 was kindly provided by Dr. Jianzhong Zhang (Chinese Disease Control and Prevention Center, Beijing, China). The *H. pylori* strains were inoculated into Brucella broth containing 5% FBS under microaerophilic conditions (5% O_2_, 10% CO_2_, and 85% N_2_) at 37 °C. For *H. pylori* infection, GC cells were seeded in six-well plates and cultured to reach 80–90% confluency with antibiotics free cell culture medium, and then the *H. pylori* was harvested, resuspended with phosphate-buffered saline (PBS) and added to the GC cells at a multiplicity of infection of 100:1. The *H. pylori*-infected GC cells were incubated for 2 or 4 h and collected.

### Statistical analysis

The investigators were randomly assigned to different groups during the experiments. The sample size chosen was based on the “3 R” principle and *n* > 3 to perform the statistical analysis. The variance is similar between the groups that are being statistically compared. Data are expressed as mean ± SD (Standard Deviation). The data were analyzed with GraphPad Prism v7.0 4 software using Student *t*-test (two-tailed) or two-way ANOVA test. All experiments were repeated independently at least three times, with similar results obtained. *p* < 0.05 was considered statistically significant.

## Results

### STYX is highly expressed in GC tissues

**Fig. 1:**
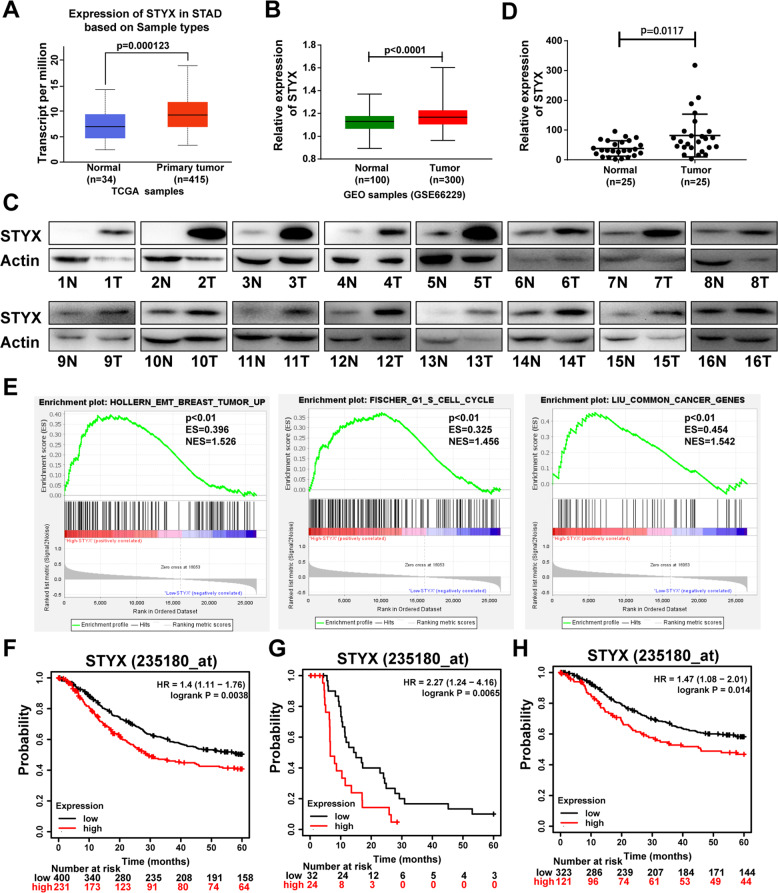
STYX is highly expressed in GC, and the expression level of STYX is associated with the prognosis of GC patients. **A** Analysis of mRNA expression level of STYX in GC tissues and non-tumor tissues according to TCGA database. **B** Analysis of mRNA expression level of STYX in GC tissues according to GEO (GSE66229) database. **C** The protein expression of STYX was detected using western blot in 25 pairs of GC tissues and the adjacent non-tumor tissues of patients (N: non-tumor tissue, T: tumor tissue). The representative results were showed. **D** The protein expression of STYX in the GC tissues and the corresponding non-tumor tissues was measured using the ImageJ software and normalized to the expression of β-actin. The data were statistically analyzed using GraphPad Prism v7.0 4 software with a paired Student *t*-test. **E** Enrichment plots of gene expression signatures for EMT, Cell cycle and common cancer genes according to STYX mRNA expression in a published cohort (http://www.cbioportal.org/, TCGA, Nature 2014). The barcode plot indexed the position of the genes in each gene set. Red and blue colors indicated high and low level of STYX. ES enrichment score, NES normalized enrichment score. **F** The Kaplan–Meier Plotter database analyzed the overall survival (OS) of GC patients in relation to the STYX levels. **G** The Kaplan–Meier Plotter database analyzed survival of GC patients with lymph node metastasis. **H** The Kaplan–Meier Plotter database analyzed survival of GC patients without lymph node metastasis.

To investigate the expression of STYX in gastric tissues, we used TCGA and GEO (GSE66229) datasets to analyze the expression of STYX mRNA in GC tissues and the non-tumor tissues and found that STYX was obviously upregulated in GC tissues (Fig. 1A-B). We further used western blot to check the protein level of STYX in GC tissues and the adjacent noncancerous tissues. We found that STYX expression was upregulated in 68% (17/ 25) of the GC tissues (Fig. 1C). We then quantified the western blot band with ImageJ software and statistically analyzed the data with paired *t*-test. The results showed that the expression level of STYX is significantly higher in GC tissues than that in the adjacent non-tumor tissues (*p* = 0.0117) (Fig. 1D). From the result of GSEA (gene set enrichment analysis) in a published GC cohort (http://www.cbioportal.org/, TCGA, Nature 2014), we found that STYX was significantly associated with EMT pathway (*p* < 0.01), cell cycle (*p* < 0.01), and common cancer gene set (*p* < 0.01) (Fig. 1E). Kaplan–Meier Plotter Database (235180_at) analysis of the overall survival and survival with or without lymph node metastasis of GC patients indicated that higher expression of STYX in GC tissues caused a shorter survival period (Fig. 1F-H).

### STYX facilitated the proliferation and migration of GC cells

**Fig. 2:**
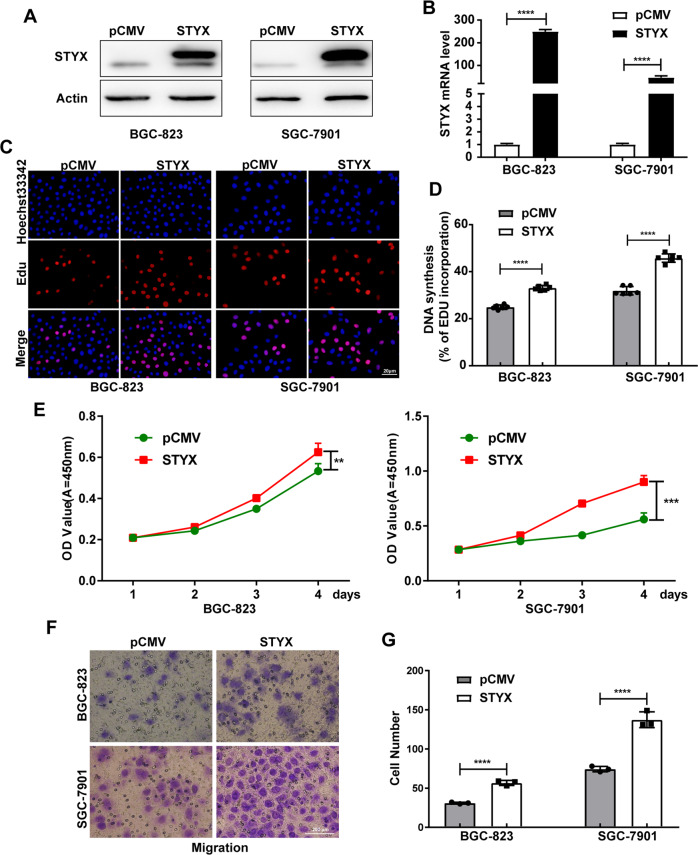
STYX overexpression promotes GC cells proliferation and migration in vitro. **A** Western blot analysis of STYX protein level in GC cells transfected with the control vector (pCMV) or STYX overexpression vector (STYX). **B** qRT-PCR analysis of STYX mRNA level in transfected GC cells. **C** EdU analysis of the cell proliferation ability in transfected GC cells. The representative results were showed. **D** Statistical analysis of the EdU-positive cell ratio in GC cells. The data are expressed as the means ± SD from six independent experiments. **E** CCK-8 analysis of the cell proliferation ability in transfected GC cells. The data are expressed as the means ± SD from three independent experiments. **F** Transwell migration assay in transfected GC cells. The representative results were showed. **G** Statistical analysis of the cell numbers passing through the transwell filter in GC cells. The data are expressed as the means ± SD from three independent experiments. The data shown in **D** and **G** were analyzed using GraphPad Prism v7.0 4 software with a nonpaired Student *t*-test. The data shown in **E** were analyzed using a two-way ANOVA test. **p < 0.01; ***p < 0.001; ****p < 0.0001.

To investigate the biological role of STYX in GC cells, we constructed the overexpression vector of STYX (pCMV-STYX) and transfected the overexpression vector or the control vector into the GC cells. The transfection efficiency was verified by western blot and qRT-PCR (Fig. 2A-B). The proliferation ability of GC cells was detected with EdU and CCK-8 assay and the migration ability of GC cells was determined with Transwell assay. Our results demonstrated that enforced expression of STYX enhanced the proliferation and migration ability of GC cells significantly (Fig. 2C-G).

To further determine whether STYX knockdown has the opposite effect, we designed two siRNAs to specifically target STYX and transfected them into HGC-27 and SGC-7901 cells. qRT-PCR and western blot results validated that STYX expression level can be inhibited efficiently by the STYX siRNAs (Fig. S1A and B). EdU, CCK-8, and Transwell assays results showed that the proliferation and migration ability of GC cells was suppressed significantly by STYX siRNAs in GC cells (Fig. S1C-G).

### STYX promoted the growth and metastasis of GC cells in vivo

**Fig. 3:**
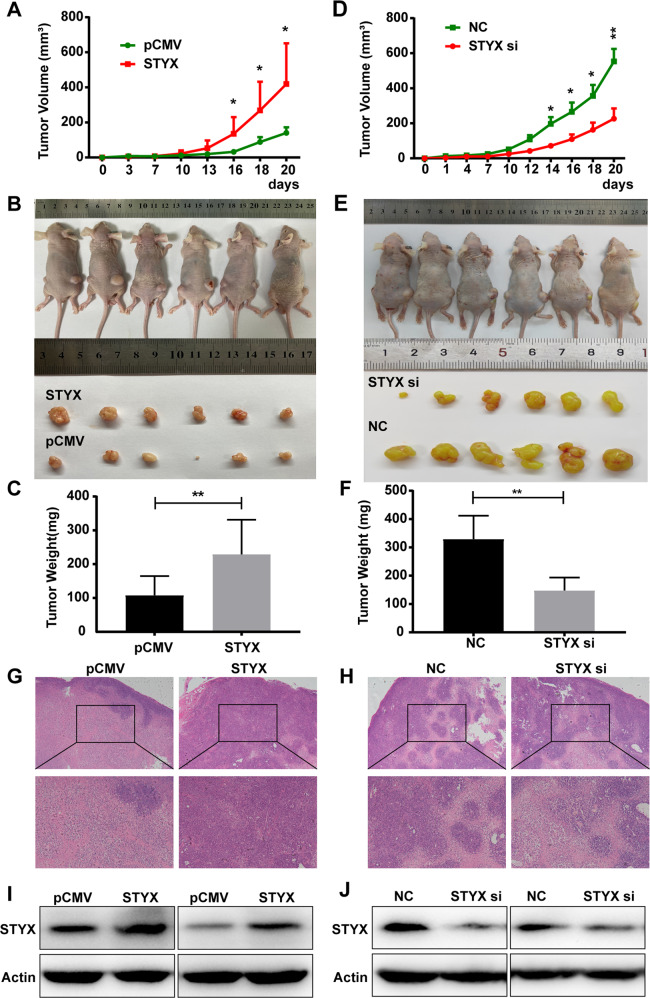
STYX affects the tumor growth of GC in vivo. **A** The nude mice were subcutaneously injected with BGC-823 cells transfected with pCMV (left) or STYX overexpression vector (STYX) (right) in the two flanks of the mice. After 3 days, the subcutaneous tumor size was measured every 3 day or 2 day and the tumor volume was calculated. Statistical analysis of the tumor volume in the mice using GraphPad Prism v7.0 4 software with a paired Student *t*-test. The data are expressed as the means ± SD. **B** The tumor-bearing mice and the dissected tumors were shown. A ruler was used to indicate the size of the tumors. **C** Statistical analysis of the tumor weight in mice injected with GC cells transfected with the control vector or STYX overexpression vector using GraphPad Prism v7.0 4 software with a paired Student *t*-test. Data were the means ± SD. **D** The tumor volume was measured and calculated in the nude mice which were subcutaneously injected with BGC-823 cells transfected with negative control (right) or stable knockdown of STYX (STYX si) (left) in the two flanks of the mice. Statistical analysis of the tumor volume in the mice using GraphPad Prism v7.0 4 software with a paired Student *t*-test. The data are expressed as the means ± SD. **E** The tumor-bearing mice and the dissected tumors were photographed and shown. A ruler was used to indicate the size of the tumors. **F** Statistical analysis of the tumor weight in mice injected with different cells. Data were the means ± SD. **G, H** Hematoxylin and eosin (HE) staining was used to detect the tumors. **I, J** Western blot analysis of the expression of STYX in the tumors. Representative results were shown.

To investigate the effects of STYX on tumorigenesis in vivo, we injected the GC cells with STYX overexpression or knockdown into the subcutaneous of the nude mice. Our results indicated that the volume and weigh of the xenografts in STYX overexpression group was much larger and heavier than those in the NC group (Fig. 3A-C). On the contrary, the tumor volume and tumor weigh in STYX knockdown group were significantly smaller and lighter than those in the NC group (Fig. 3D-F). HE staining validated that all tumors were solid tumors (Fig. 3G-H). Western blot results indicated that the expression of STYX was increased in the tumors from STYX overexpression group, whereas STYX expression was decreased in the tumor from STYX knockdown group compared with the control group (Fig. 3I-J).

**Fig. 4:**
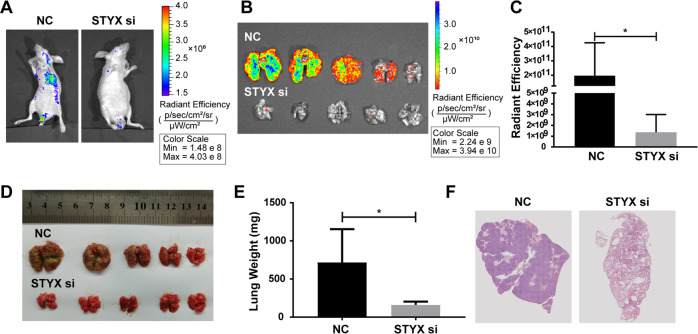
STYX affects the tumor metastasis of GC in vivo. **A, B** BGC-823 cells with negative control (NC) or stable knockdown of STYX (STYX si) were injected into the tail vein of the nude mice. The effect of STYX knockdown on the lung tumor metastasis was detected by bioluminescent images. Representative images of the whole mice or the lung were shown. **C** Quantification and statistical analysis of the bioluminescence imaging in the lung region. The data are expressed as the means ± SD. **D** Representative images of lung metastasis were shown. **E** Statistical analysis of the lung weight of mice injected with different cells. Data were expressed as the means ± SD. **F** H&E staining of lung tissues was used to detect the metastasis nodules. Representative images were shown.

To further explore the effect of STYX on the tumor metastasis of GC cells in vivo, we injected GC cells of stable luciferase-labeled control or STYX knockdown into nude mice through the tail vein and then examined the luminescence signals of the mice for tumor cell colonization. As shown in Fig. 4A-C, the bioluminescence intensity in STYX knockdown group was much lower than that in the control group. We then weighed the lungs of the mice. The results showed that the lungs from the mice injected with STYX knockdown cells were significantly smaller and lighter than those from mice injected with control cells (Fig. 4D-E). Additionally, H&E staining revealed that the mice injected with STYX knockdown cells formed fewer metastatic nodules in the lungs (Fig. 4F). Together, these data strongly indicated that STYX promoted GC growth and metastasis in vivo.

### STYX interacts with FBXO31 and inhibits FBXO31 function in GC cells

**Fig. 5:**
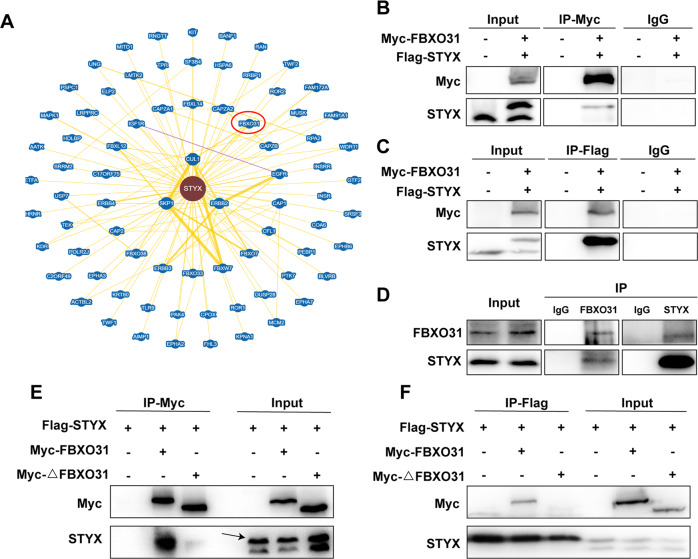
STYX interacts with FBXO31. **A** The interaction between STYX and FBXO31 was found through the analysis of Biogrid database (https://thebiogrid.org/). **B, C** Myc-FBXO31 or Flag-STYX were co-transfected into SGC-7901 cells for 48 h. Co-immunoprecipitation was performed with either Myc antibody to probe for STYX by anti-STYX antibody or Flag antibody to probe for FBXO31 by anti-Myc antibody. Immunoglobulin (IgG) was used as a control. **D** Endogenous interaction between FBXO31 and STYX protein was detected by co-immunoprecipitation with the FBXO31 or STYX antibody in 293 T cells and western blot were performed to detect the co-precipitated STYX or FBXO31. IgG was used as a control. **E, F** pCMV or Myc-FBXO31 or Myc-FBXO31ΔF and Flag-STYX were co-transfected into SGC-7901 cells for 48 h. Co-immunoprecipitation was performed with either Myc antibody to probe for STYX by anti-STYX antibody or Flag antibody to probe for FBXO31 by anti-Myc antibody.

To explore the potential mechanism of STYX in GC cells, we used Biogrid database to predict the interaction proteins of STYX and found that there are several F-box proteins that have the potential interaction with STYX (Fig. 5A). Among these F-box proteins, FBXO31 was downregulated in GC and involved in the tumorigenesis and development of GC [17, 18]. Therefore, we performed co-immunoprecipitation (Co-IP) assay to examine the interaction between STYX and FBXO31. Our results showed that ectopically expressed Flag-STYX was co-immunoprecipitated by Myc-FBXO31 (Fig. 5B). A similar result was obtained in a reciprocal Co-IP experiment using Flag antibody (Fig. 5C). Then we used 293T cells to carry out the endogenous IP assay and verified a physical interaction between FBXO31 and STYX (Fig. 5D). To further explore whether the interaction depends on the F-box domain of FBXO31, we co-transfected the F-box domain deletion mutation vector Myc-FBXO31ΔF and STYX expression vector Flag-STYX into GC cells and performed Co-IP experiments. The results demonstrated that the Myc-FBXO31ΔF can not co-immunoprecipitate with Flag-STYX (Fig. 5E-F). These data suggest that FBXO31 interacts with STYX in GC cells, and the F-box domain of FBXO31 is essential for the interaction.

**Fig. 6:**
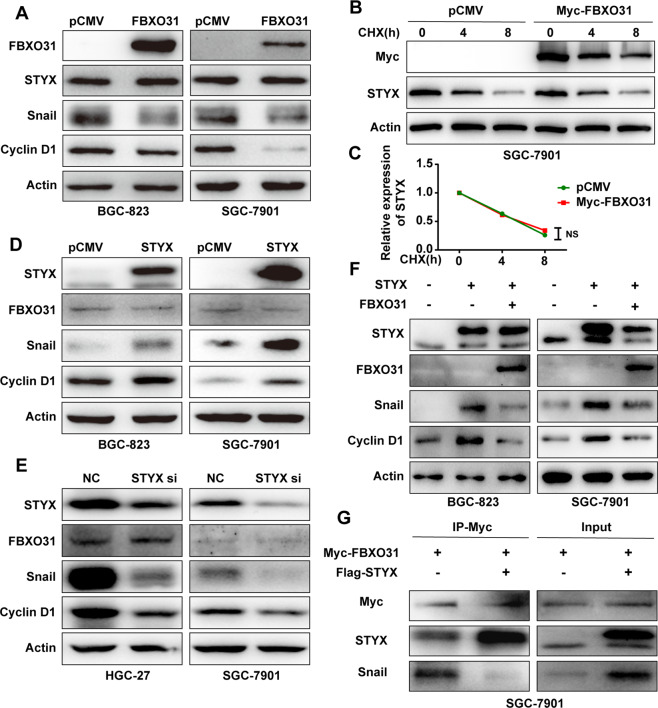
STYX influences the progression of GC by interacting with FBXO31. **A** Western blot was used to detect STYX, Snail1 and CyclinD1 in GC cells transfected with pCMV or FBXO31. **B** The GC cells were transfected with pCMV and FBXO31 expression vector. Then the transfected cells were treated with CHX at indicated time. Western blot analysis of the expression of STYX in the treated GC cells. **C** STYX expression levels were quantified by measuring the band intensities using “ImageJ” software. The values were normalized to β-actin. **D** Western blot was used to detect FBXO31, Snail1, and CyclinD1 in GC cells transfected with STYX expression vector. **E** Western blot was used to detect FBXO31, Snail1, and CyclinD1 in GC cells transfected with STYX siRNA. **F** Western blot was used to detect Snail1 and CyclinD1 in GC cells co-transfected with STYX and FBXO31. **G** Myc-FBXO31 and Flag-STYX were co-transfected into SGC-7901 cells for 48 h. Co-immunoprecipitation was performed with Myc antibody to detect STYX or Snail1 level.

Since FBXO31 is the substrate recognition component of Skp1/Cul1/F-box (SCF) E3 ubiquitin ligase complex and mediates the ubiquitination and degradation of the target substrates [20, 21], we asked whether STYX is a substrate for FBXO31. We transfected FBXO31 overexpression vector into the GC cells and determined the expression of STYX with western blot. Our results showed that FBXO31 overexpression has no detectable effect on the expression of STYX, but decreased the expression level of CyclinD1 and Snail1, which are the well-characterized substrates for FBXO31 (Fig. 6A). To further confirm this findings, we determined the effect of FBXO31 on the turnover rate of the STYX by adding CHX into the transfected GC cells. The results showed that the turnover of STYX was not affected by FBXO31 to any detectable extent (Fig. 6B-C). Taken together, we conclude that STYX is unlikely to be a substrate for FBXO31.

We then tested whether STYX regulated the expression and function of FBXO31. We transfected STYX overexpression vector or siRNAs into the GC cells and then detected the levels of FBXO31 and its substrates CyclinD1 and Snail1. The results showed that STYX overexpression or knockdown has no effect on the expression of FBXO31, but increased or decreased the expression level of FBXO31 substrates, CyclinD1 and Snail1 (Fig. 6D-E). qRT-PCR results showed that STYX overexpression or knockdown has no effect on the mRNA levels of CyclinD1 and Snail1 (data not show), indicating that the regulation occurs at the post-transcriptional level. We further determined whether the effect of STYX on cyclinD1 and Snail1 was dependent on FBXO31. As shown in Fig. 6F, STYX-mediated regulation on CyclinD1 and Snail1 can be reversed by the co-expression of STYX and FBXO31. Furthermore, we determined whether STYX affected the interaction between FBXO31 and its substrate. The Co-IP results showed that STYX overexpression decreased the interaction between FBXO31 and its substrate Snail (Fig. 6G). These findings indicate that STYX acts as a negative regulator of FBXO31 function.

### STYX regulated GC cell proliferation and migration via FBXO31

**Fig. 7:**
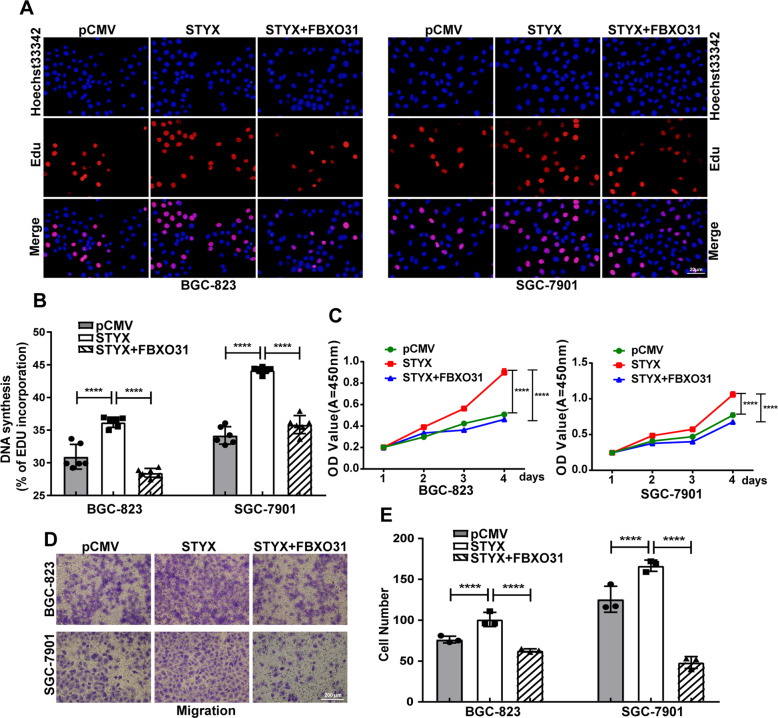
STYX-mediated promotion of GC cells proliferation and migration can be abrogated by FBXO31. **A** EdU analysis of the cell proliferation ability in GC cells with different transfection. The representative results were showed. **B** Statistical analysis of the EdU-positive cell ratio in transfected GC cells. The data are expressed as the means ± SD from six independent experiments. **C** CCK-8 analysis of the cell proliferation ability in transfected GC cells. The data are expressed as the means ± SD from three experiments. **D** Transwell invasion and migration assay in transfected GC cells. **E** Statistical analysis of the cell numbers passing through the transwell filter in GC cells. The data are expressed as the means ± SD from three experiments. The data shown in **B** and E were analyzed using GraphPad Prism v7.0 4 software with a nonpaired Student *t*-test. The data shown in **C** were analyzed using a two-way ANOVA test. **p < 0.01; ***p < 0.001; ****p < 0.0001.

To further investigate whether FBXO31 was involved in STYX-mediated biological behaviors on GC cells, we performed the rescue experiments. EdU and CCK-8 were used to detect the cell proliferation ability. Transwell assay was used to determine the cell migration ability. As shown in Fig. 7, overexpression of STYX significantly increased the cell proliferation and migration of GC cells, while co-overexpression of STYX and FBXO31 significantly abrogated STYX-mediated biological function.

### *H.pylori* infection promotes STYX expression via c-Jun

**Fig. 8:**
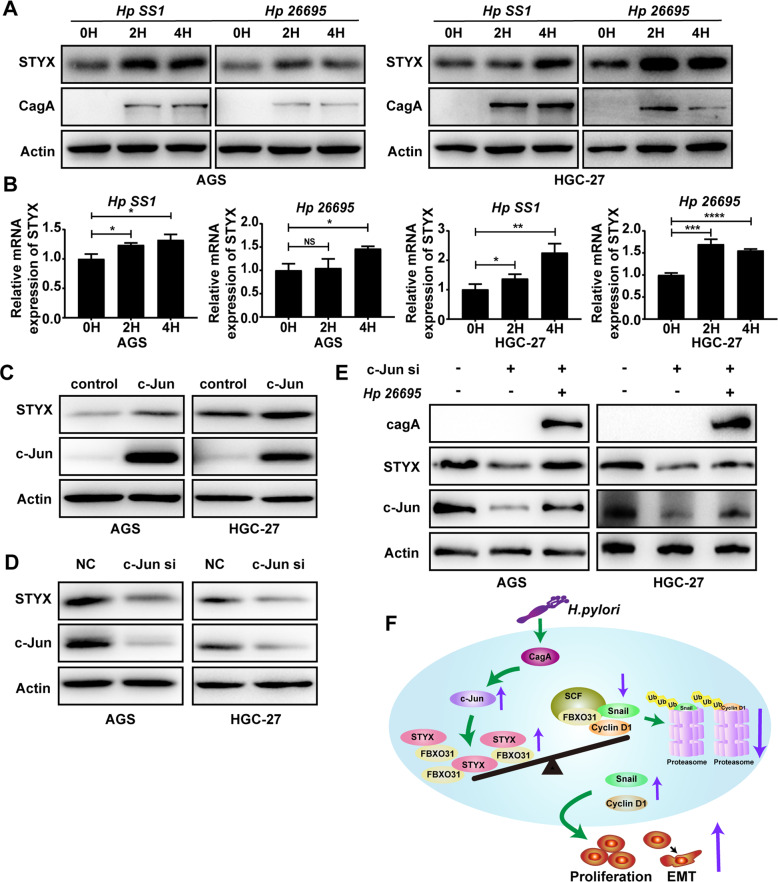
*H.pylori* infection promotes STYX expression via c-Jun. **A** Western blot analysis of STYX protein expression in AGS and HGC-27 cells infected with *H.pylori* (SS1 or 26695) at different time points. **B** qRT-qPCR analysis of STYX mRNA expression in GC cells infected with *H. pylori* at different time points. **C** Western blot was used to detect STYX transfected with c-Jun expression vector. **D** Western blot was used to detect STYX transfected with c-Jun siRNA. **E** Western blot was used to detect STYX in GC cells co-transfected with c-Jun siRNA and H.pylori (26695). **F** Working model for STYX promoting carcinogenesis and metastasis of GC.

Since *H. pylori* infection is considered to be the strongest risk factor for GC [22, 23], we wonder whether *H.pylori* infection affected the expression of STYX. We infected the GC cells with *H. pylori* 26695 and SS1 for 2 and 4 h and determined the expression of STYX with qRT-PCR and western blot. The results showed that both STYX protein and mRNA level was significantly increased with *H. pylori* infection in GC cells (Fig. 8A-B). Then, we used Jaspar database to predict the transcription factors that can bind to STYX promoter and found that there are several conserved c-Jun-binding elements in STYX promoter. Besides, it has been reported that *H.pylori* infection promotes c-Jun expression [24, 25]. Therefore, we asked whether *H.pylori* promotes STYX expression via c-Jun. We transfected the expression vector or siRNA of c-Jun into the GC cells and found that c-Jun overexpression dramatically increased STYX expression, while c-Jun siRNA decreased STYX expression (Fig. 8C-D). Furthermore, when we knockdown c-Jun with siRNA, *H.pylori-*mediated upregulation of STYX can be abrogated (Fig. 8E).

## Discussion

It has been recently reported that the pseudophosphatase STYX plays oncogenic roles in several tumors, including colorectal cancer, breast cancer, and endometrial cancer [8, 10, 11]. However, the expression, biological roles and regulatory mechanism of STYX in GC remain largely unknown. In the present study, we found that STYX was highly expressed in GC tissues compared with the adjacent non-tumor tissues and the high expression level of STYX was significantly associated with poorer 5-year OS (Overall survival) in the GC patients. Gene set enrichment analysis (GSEA) showed that STYX expression was significantly associated with EMT pathway, cell cycle, and common cancer gene set. This is the first report about the expression level of STYX and its correlation with the prognosis of GC patients. We further investigated the effects of STYX on GC and found that overexpression of STYX increased GC cells proliferation and migration in vitro. Nude mice in vivo experiments verified the tumor-promoting activity of STYX. In contrast, STYX knockdown had the opposite effects on GC cells and inhibited the tumor growth and lung metastasis in vivo. These results validated the oncogenic role of STYX in GC and demonstrated that STYX may serve as a valuable diagnostic and prognostic marker for GC patients.

Previous studies from Reiterer et al. showed that STYX can bind to the F-box domain of FBXW7 in breast cancer, thereby preventing FBXW7 from the formation of SCF^FBXW7^ complex and further inhibiting FBXW7 activity to degrade its target substrates [8]. He et al. also reported STYX interacted with FBXW7 and inhibited the function of FBXW7 in colorectal cancer [10]. Furthermore, FBXW7 expression was negatively correlated with STYX expression in CRC tissues, and low STYX levels accompanying high FBXW7 levels predicted favorable prognosis of CRC patients [10]. We wondered whether STYX can bind to other F-box proteins in GC. We used Biogrid database to identify the interaction proteins of STYX and found that FBXO31 has the potential possibility to interact with STYX. Furthermore, the screen results of the liquid chromatography coupled with tandem mass spectrometry (LC-MS/MS) from Reiterer et al. also exhibited that FBXO31 may be one of the potential F-box proteins to interact with STYX [8]. Our previous studies demonstrated that FBXO31 functions as a tumor suppressor in GC by targeting several important oncoproteins, such as CyclinD1 [17], Snail1 [18], and the loss of FBXO31 protein is associated with a higher malignant phenotype and poorer prognosis. As for the regulatory mechanism, previous studies mainly focused on the regulation of FBXO31 expression by miRNAs [17]. In the current work, we provide evidences to indicate that STYX represses FBXO31 functions in GC cells by direct protein–protein interaction without changing its expression: (1) STYX interacts with FBXO31 in GC cells, and the interaction is dependent on the F-box domain of FBXO31. Therefore, STYX overexpression inhibits the interaction of FBXO31 with its substrate Snail1. (2) Overexpression or knockdown of STYX has no effect on FBXO31 expression, but significantly decreased or increased the expression level of FBXO31 substrates, CyclinD1 and Snail1; (3) The expression changes of CyclinD1 and Snail1 mediated by STYX overexpression alone can be reversed by the co-expression of STYX and FBXO31. Given that the important roles of FBXO31 substrates, such as CyclinD1, Snail1 in tumors, we propose that the interaction of STYX-FBXO31 may represent a valuable therapeutic target for GC.

We further explored whether FBXO31 is involved in STYX-mediated biological effects of GC cells and found that the biological behaviors of GC cells mediated by STYX overexpression alone can be reversed by the co-expression of STYX and FBXO31, indicating FBXO31 may serve as a critical target for STYX in GC cells. From these results, we concluded that the biological role of STYX on GC cells was achieved by regulating FBXO31 function.

It is well-known that *H. pylori* infection contributed to the chronic gastritis and peptic ulcer, and is considered to be the strongest risk factor for the development of gastric-related malignancies [20]. *H. pylori* infection can promote the proliferation and metastasis of GC cells [26]. We wondered whether *H.pylori* infection contributed to the aberrant expression of STYX in GC. Therefore, we determined STYX expression in GC cells in the presence of *H.pylori*. Our results indicated that *H.pylori* infection obviously increased the expression of STYX. We further identified the factor that participates in *H.pylori*-mediated upregulation of STYX and found that transcription factor c-Jun plays an important role in mediating the regulation of *H.pylori* to STYX.

In conclusion, we found for the first time that STYX was aberrantly overexpressed in GC tissues and STYX expression level was negatively associated with the prognosis of GC patients. *H.pylori* infection contributed to the aberrant expression of STYX. STYX plays important roles in gastric tumorigenesis and development by interacting with FBXO31 and inhibiting the degradation of FBXO31 to its target substrates. We illustrated these findings in Fig. 8F. These findings suggested that STYX might serve as a novel prognostic biomarker and a promising therapeutic target for GC. Future studies may be needed to develop new small molecular compounds to destroy the interaction of STYX with FBXO31, thereby recovering the tumor suppressor effects of FBXO31 in GC.

## Supplementary information

Aj-checklist

Figure S1 legend

Figure S1

Table S1

Original Data File

Original Data File

Original Data File

Original Data File

Original Data File

## Data Availability

All data generated or analyzed during this study are included in this published article and its supplementary files.
