# Supplementary material for: Pseudophosphatase STYX is induced by Helicobacter pylori and promotes gastric cancer progression by inhibiting FBXO31 function
Source: Cell Death Dis. 2022 Mar 25;13(3):268. doi: 10.1038/s41419-022-04696-x (PMC8956710; doi:10.1038/s41419-022-04696-x)
Supplement: Supplementary file 2 — Figure S1 legend [file 41419_2022_4696_MOESM2_ESM.docx]

Figure.S1 STYX knockdown inhibits GC cells proliferation and migration *in vitro*. A. qRT-PCR was used to analyze STYX mRNA level in GC cells transfected with the negative control siRNA or STYX siRNAs. The data were expressed as the means ± SD from three experiments. B. Western blot analysis of STYX protein level in the cells transfected with the negative control siRNA or STYX siRNAs. C. EdU analysis of the cell proliferation ability in transfected GC cells. The representative results were showed. D. Statistical analysis of the EdU-positive cell ratio in transfected GC cells. The data are expressed as the means ± SD from six independent experiments. E. CCK-8 analysis of the cell proliferation ability in transfected GC cells. The data are expressed as the means ± SD from three independent experiments. F. Transwell migration assay in transfected GC cells. The representative results were showed. G. Statistical analysis of the cell numbers passing through the transwell filter in GC cells. The data are expressed as the means ± SD from three experiments.The data shown in D and G were analyzed using GraphPad Prism v7.0 4 software with a non-paired Student t test. The data shown in E were analyzed using a two-way ANOVA test.**p<0.01; ***p<0.001;****p<0.0001.
