# Supplementary figures and images for: Pseudophosphatase STYX is induced by Helicobacter pylori and promotes gastric cancer progression by inhibiting FBXO31 function
Source: Cell Death Dis. 2022 Mar 25;13(3):268. doi: 10.1038/s41419-022-04696-x (PMC8956710; doi:10.1038/s41419-022-04696-x)

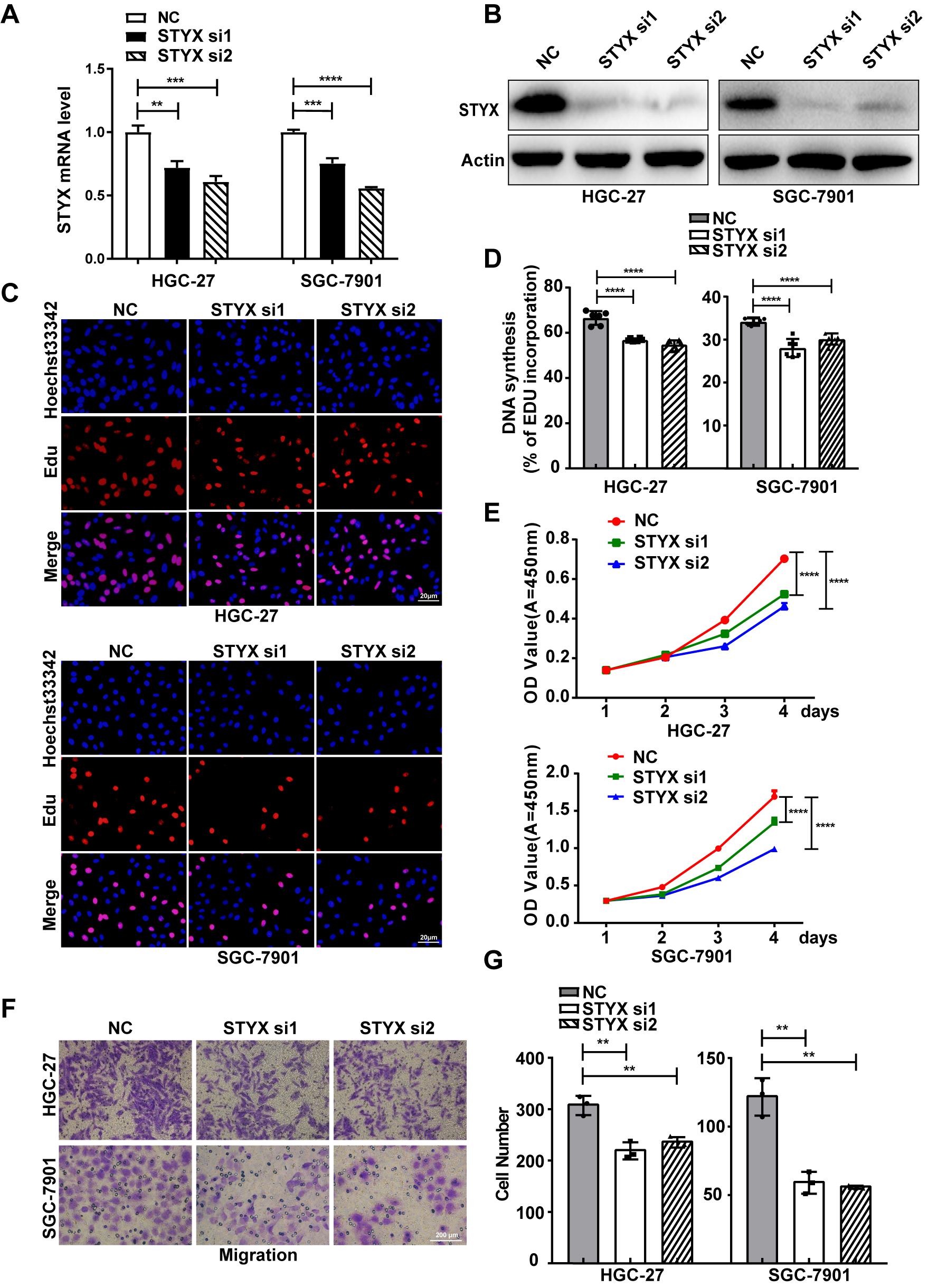

Supplement: Supplementary file 3 — Figure S1 [file 41419_2022_4696_MOESM3_ESM.tif]

Fig1 WesternBlot raw image  
STYX

Actin

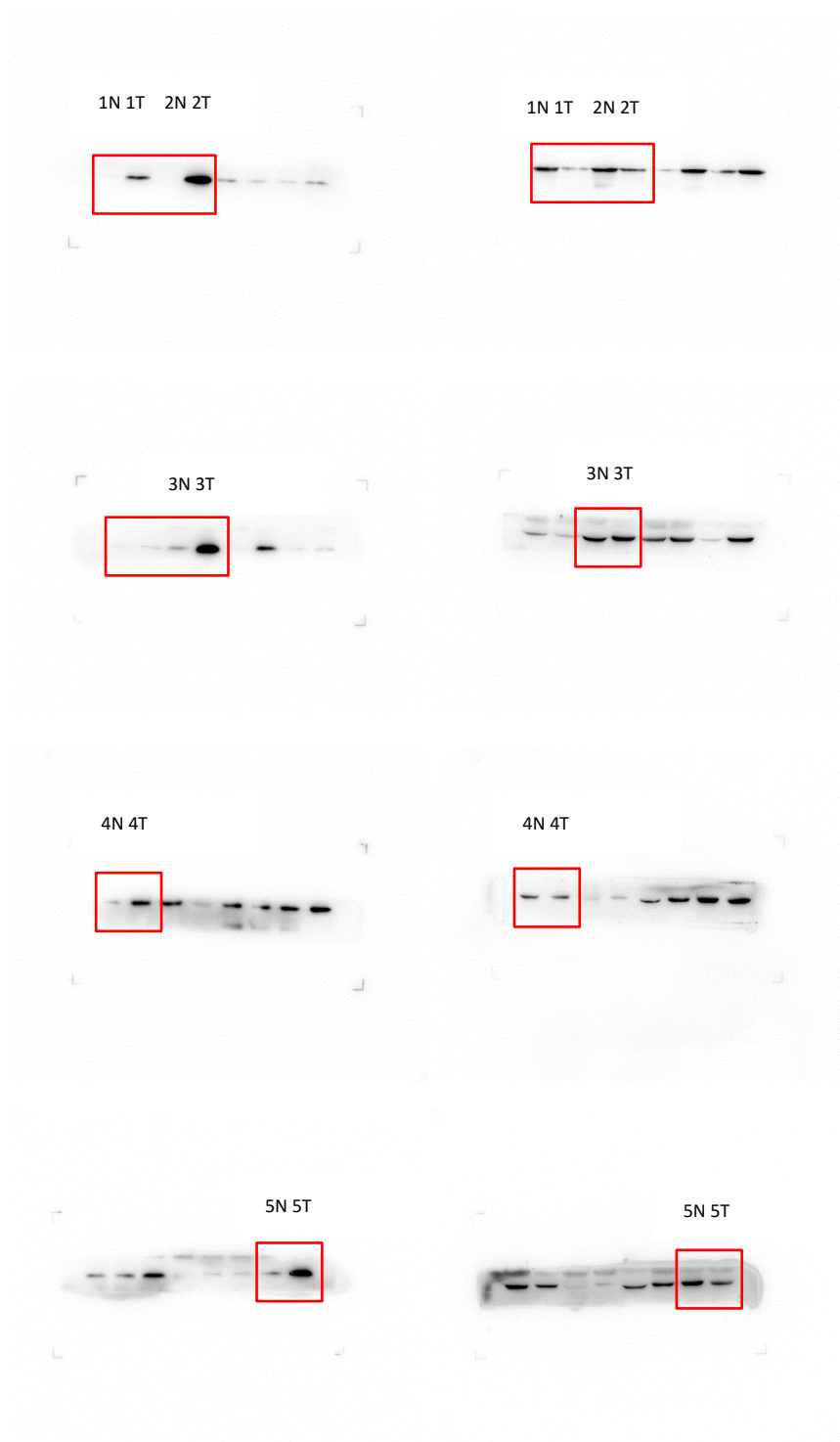

6N 6T

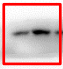

6N 6T

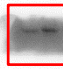

7N 7T

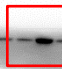

7N 7T

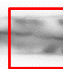

8N 8T 16N 16T

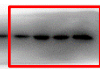

8N 8T 16N 16T

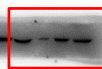

9N 9T

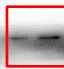

9N 9T

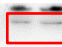

10N 10T 14N 14T

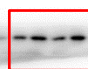

10N 10T 14N 14T

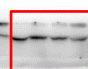

11N 11T

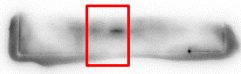

11N 11T

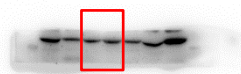

12N 12T

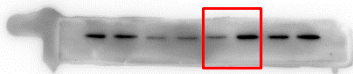

12N 12T

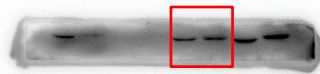

13N 13T

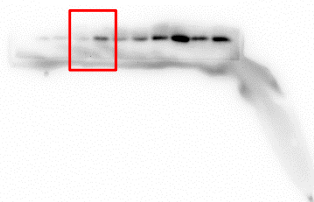

13N 13T

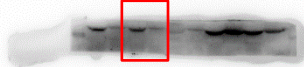

15N 15T

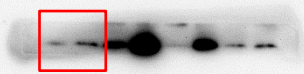

15N 15T

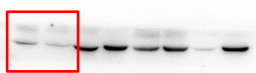

Supplement: Supplementary file 5 — Original Data File [file 41419_2022_4696_MOESM5_ESM.pdf]

Fig2 WesternBlot raw image

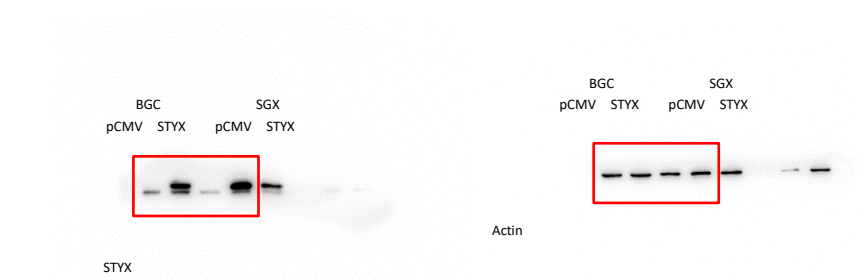

Fig3 WesternBlot raw image  
I.

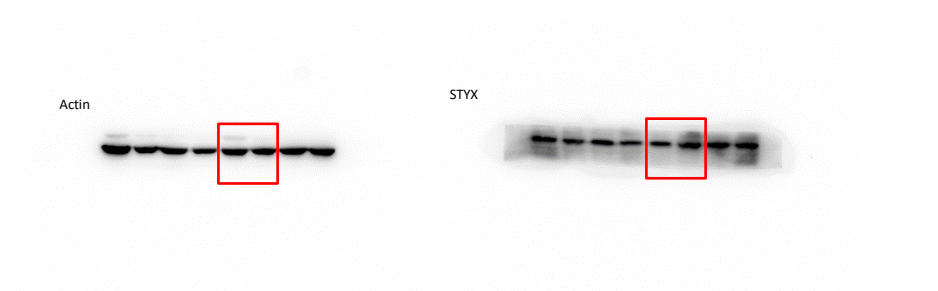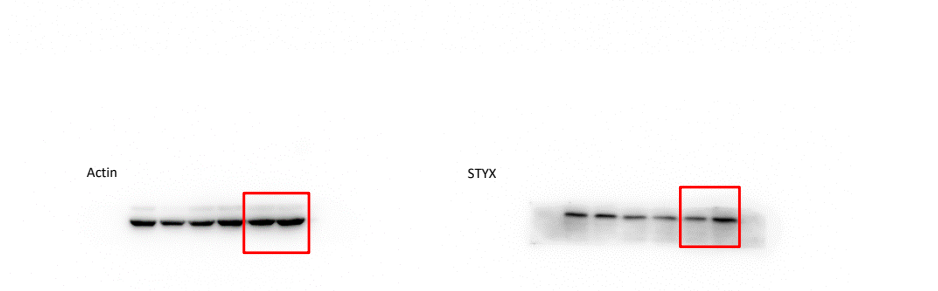

J.

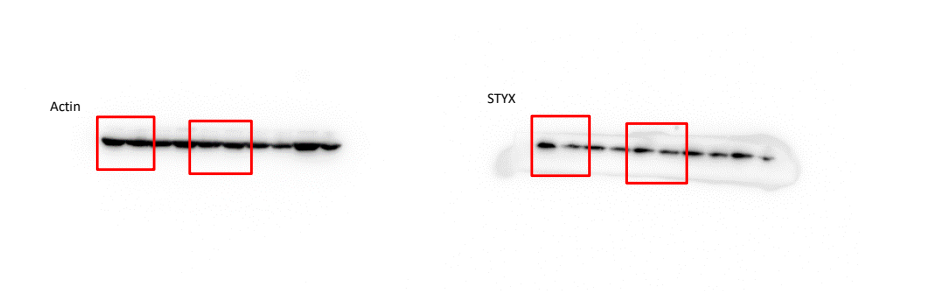

Fig5 WesternBlot raw image

B.

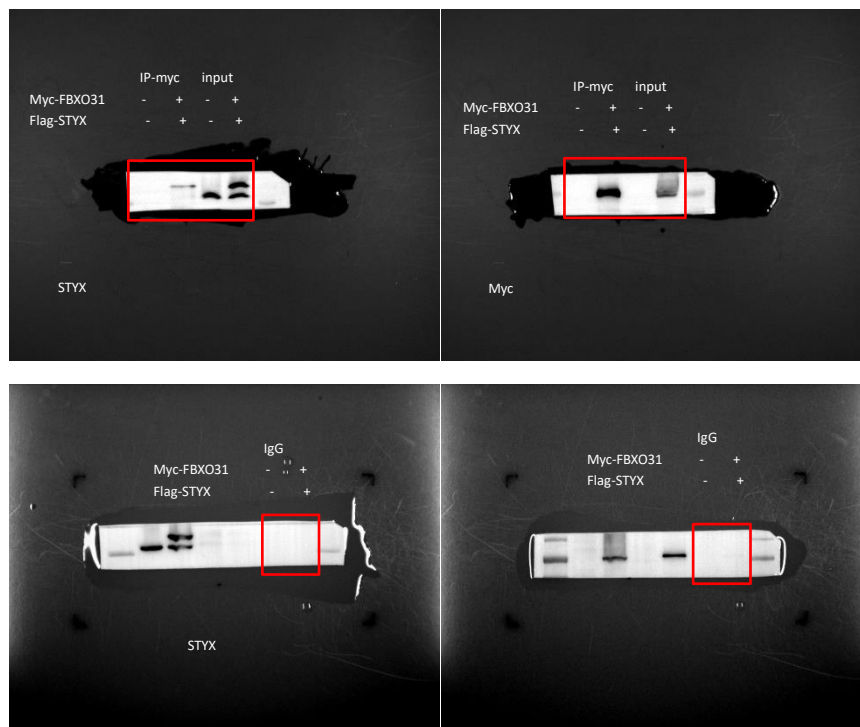

C

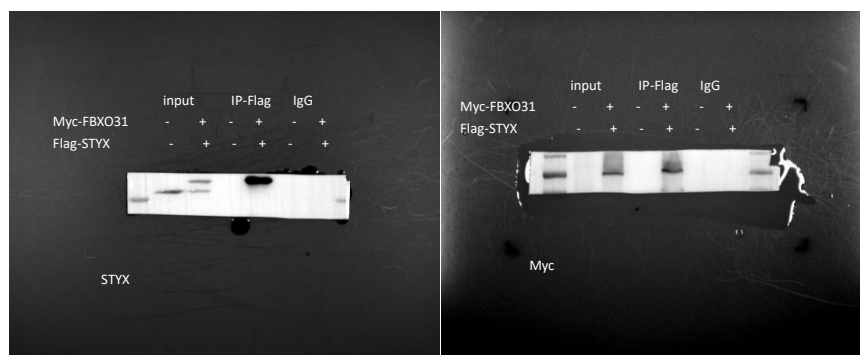

D

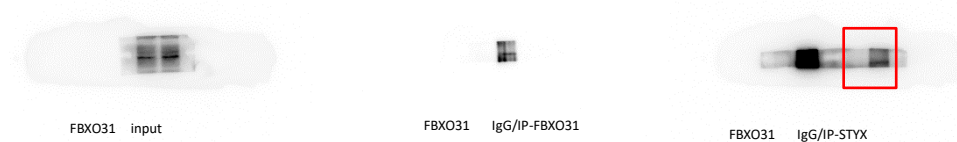

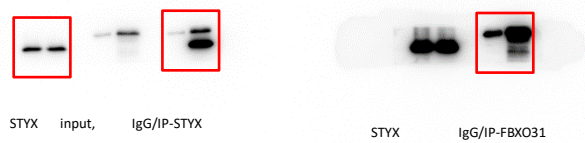

E

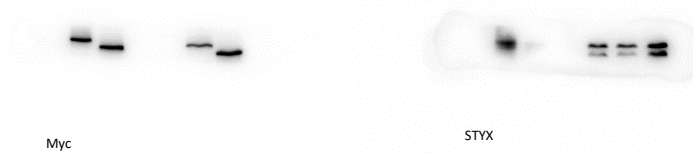

F

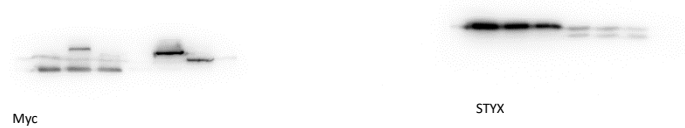

Supplement: Supplementary file 6 — Original Data File [file 41419_2022_4696_MOESM6_ESM.pdf]

Fig8 WesternBlot raw image

A. AGS SS1+26695

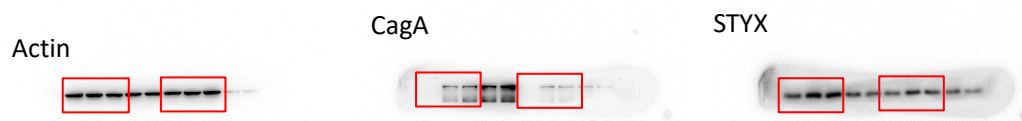

A. HGC SS1

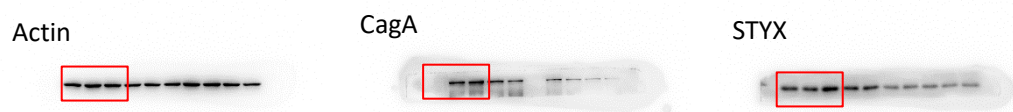

B. HGC 26695

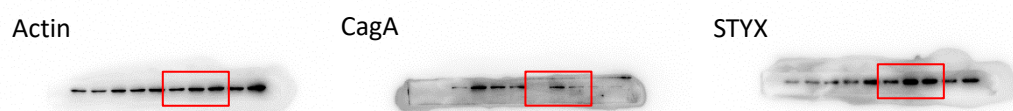

C. AGS + HGC

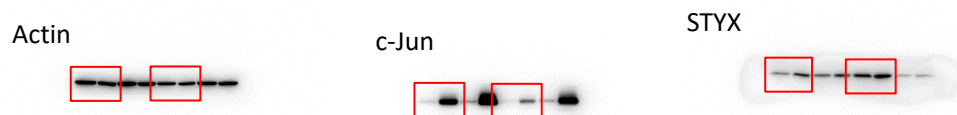

D. AGS

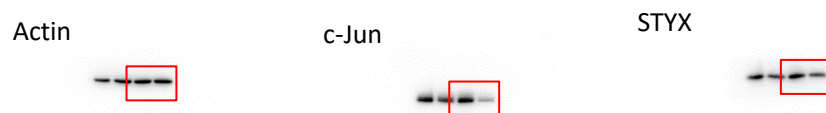

D. HGC

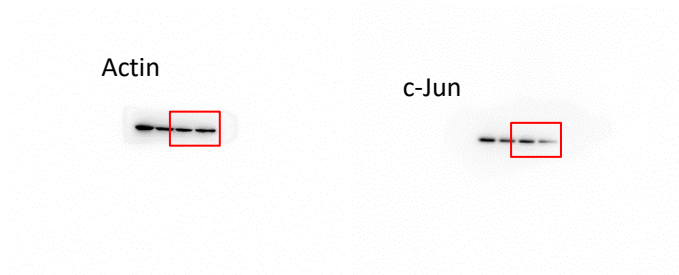

E. AGS

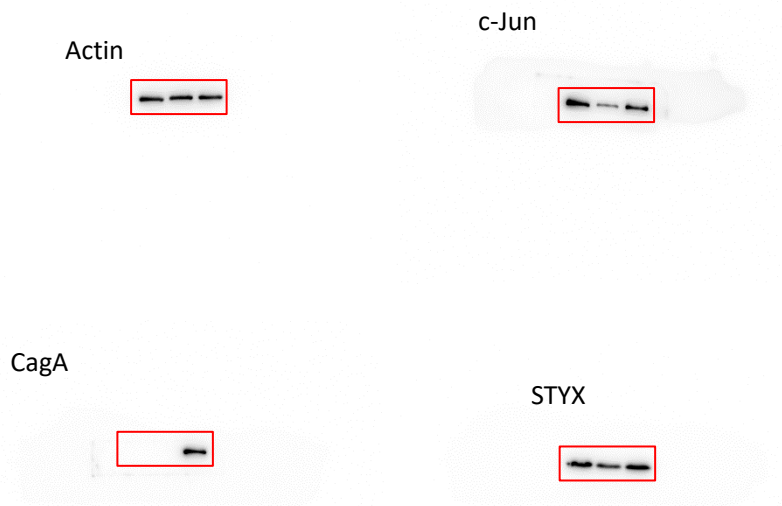

E. HGC

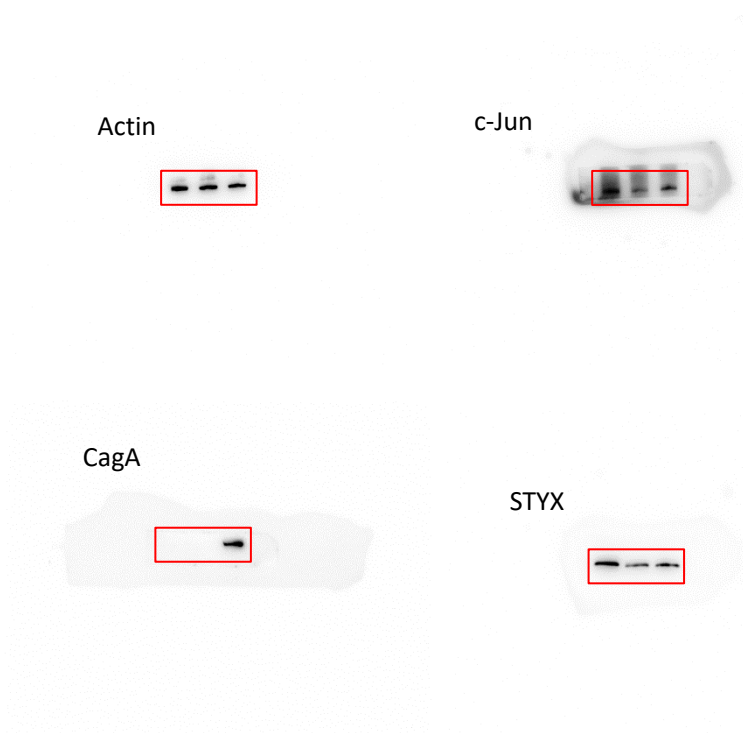

Supplement: Supplementary file 8 — Original Data File [file 41419_2022_4696_MOESM8_ESM.pdf]

FigS1 WesternBlot raw image

B. AGS

Actin

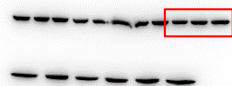

STYX

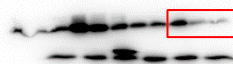

B. SGC

Actin

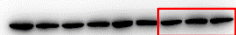

STYX

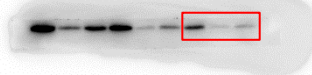

Supplement: Supplementary file 9 — Original Data File [file 41419_2022_4696_MOESM9_ESM.pdf]
