# Supplementary material for: Pseudophosphatase STYX is induced by Helicobacter pylori and promotes gastric cancer progression by inhibiting FBXO31 function
Source: Cell Death Dis. 2022 Mar 25;13(3):268. doi: 10.1038/s41419-022-04696-x (PMC8956710; doi:10.1038/s41419-022-04696-x)
Supplement: Supplementary file 4 — Table S1 [file 41419_2022_4696_MOESM4_ESM.docx]

**Table S1. Sequences for siRNA and primers**

| STYX siRNA1 | 5’-CUAUGACUAAGGAAUUUAUTT |
| --- | --- |
| STYX siRNA2 | 5’-CAUAUUCAUCUGCUAUGAATT |
| c-Jun siRNA | 5’-GACUGUAGAUUGCUUCUGU |
| Control siRNA | 5’-UUCUCCGAACGUGUCACGUTT |
| STYX forward primer for expression vector constuct | GGATCCATGGAGGACGTGAAGCTGGA |
| STYX reverse primer for expression vector constuct | CTCGAGAAGTCAGCCATTCTGTGCAG |
| STYX RT-PCR forward primer | GGAGCTTACAAATGGGAGGAAA |
| STYX RT-PCR reverse primer | TCTATCTGGAGTGGTGACATCAT |
