# Supplementary material for: Pseudophosphatase STYX is induced by Helicobacter pylori and promotes gastric cancer progression by inhibiting FBXO31 function
Source: Cell Death Dis. 2022 Mar 25;13(3):268. doi: 10.1038/s41419-022-04696-x (PMC8956710; doi:10.1038/s41419-022-04696-x)

Fig6 WesternBlot raw image

A. BGC

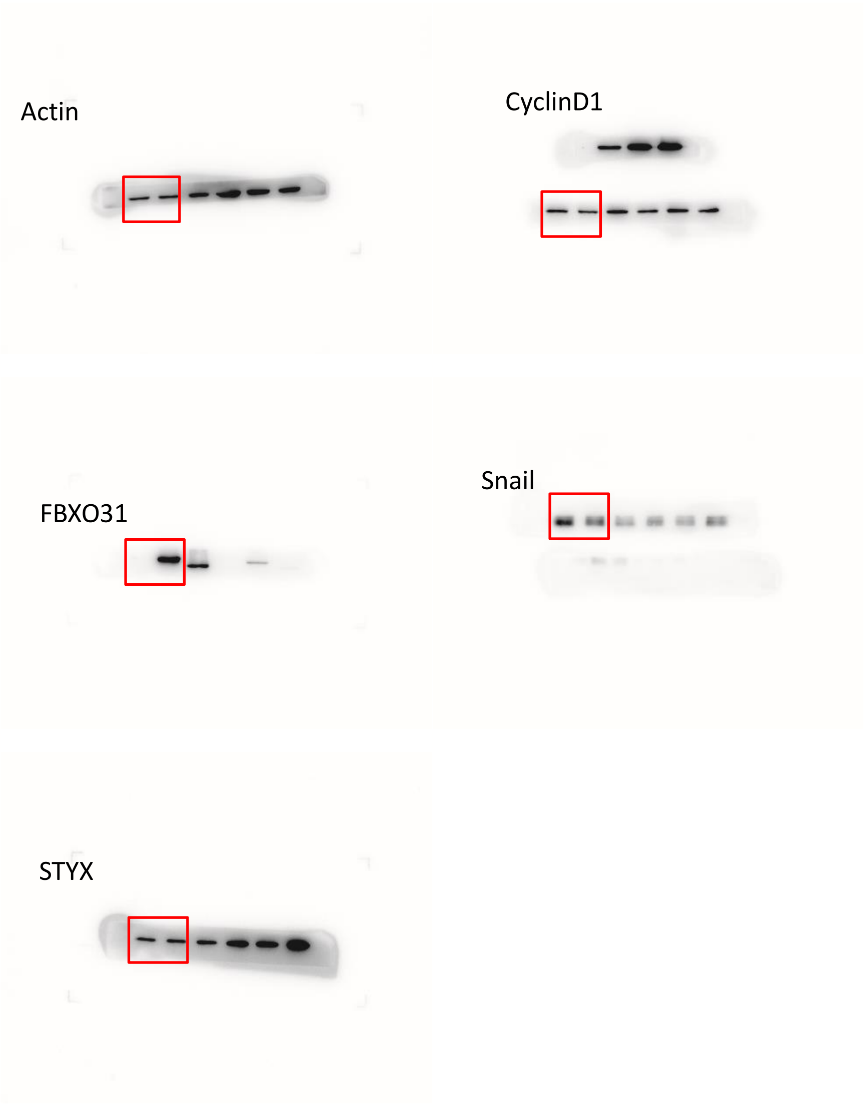

A. SGC

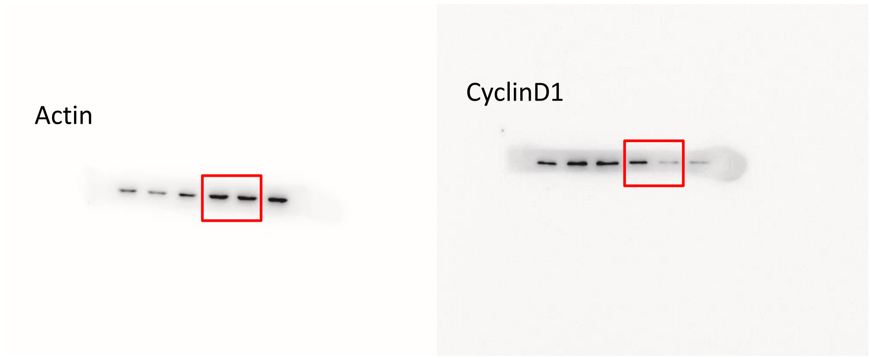

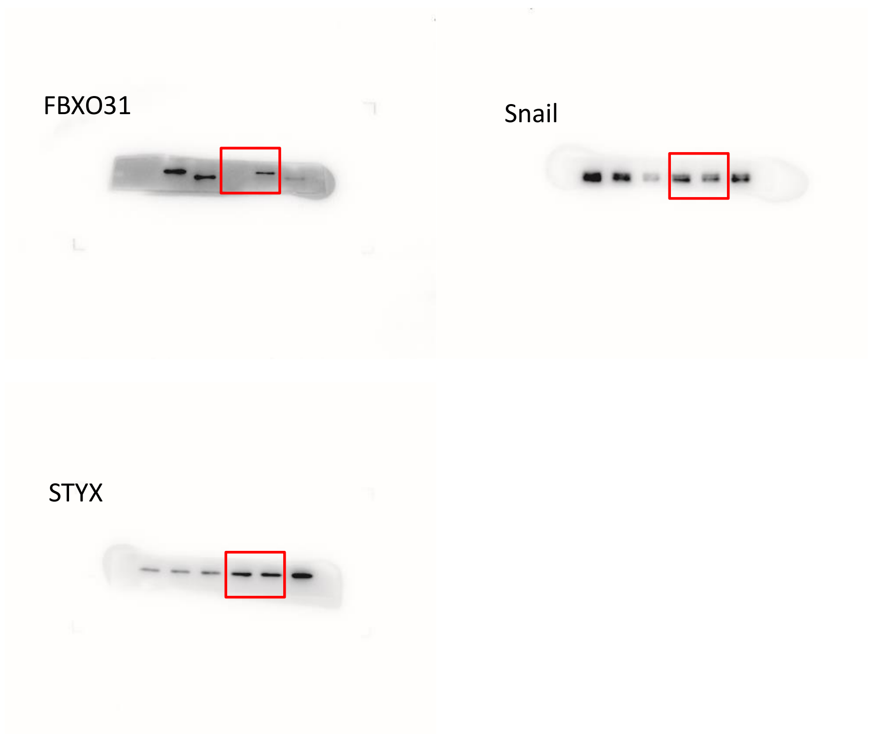

B.

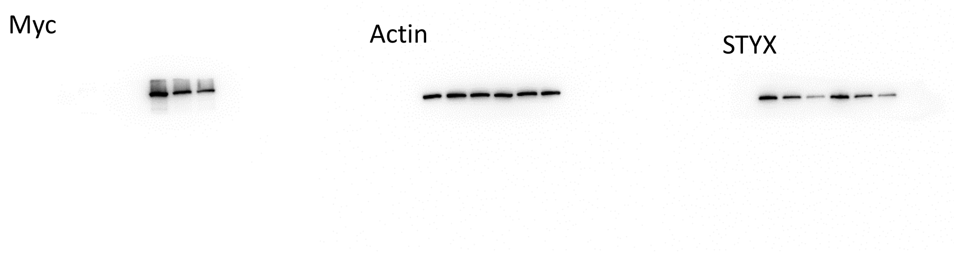

D. BGC

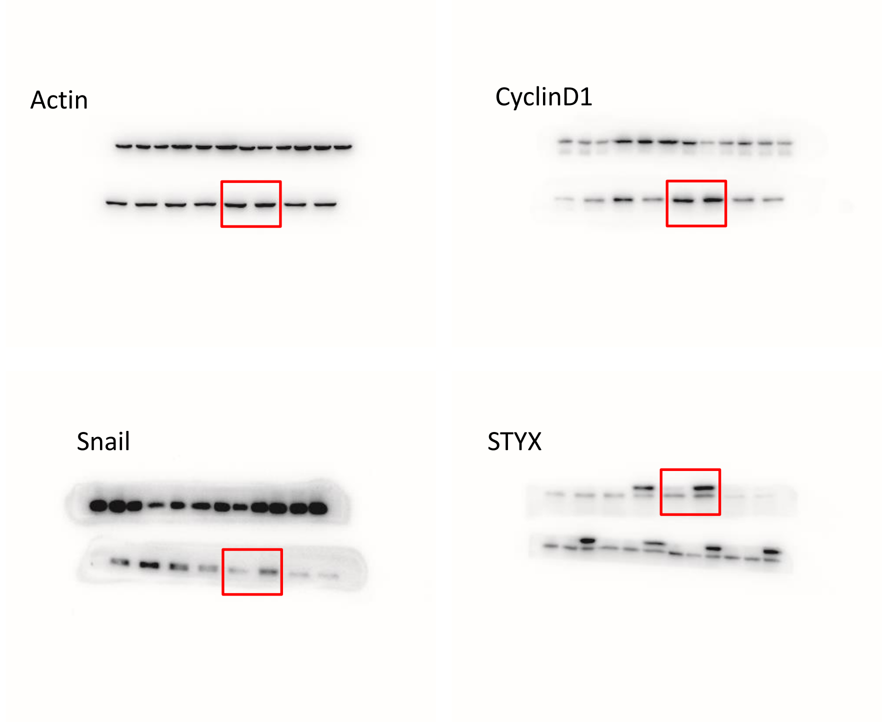

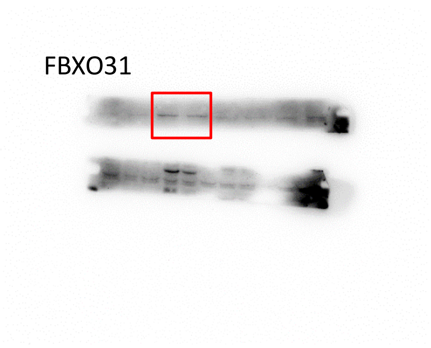

D. SGC

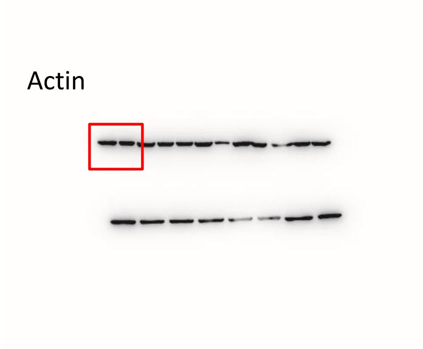

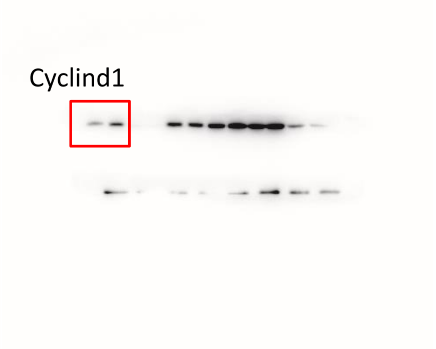

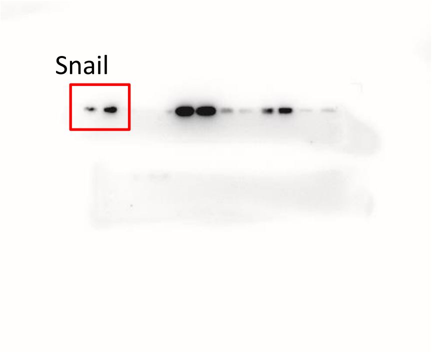

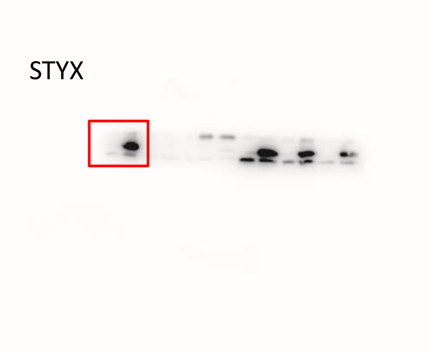

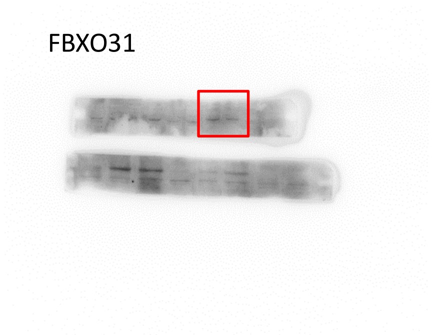

E. HGC

Actin

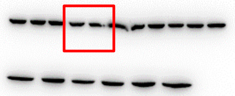

Cyclind1

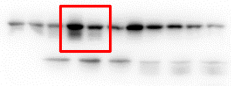

FBXO31

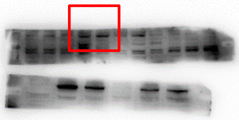

Snail

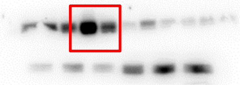

STYX

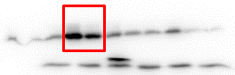

E. SGC

Actin

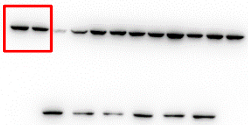

Cyclind1

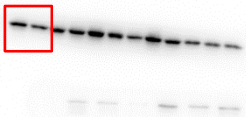

Snail

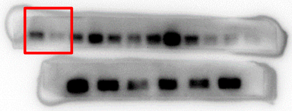

STYX

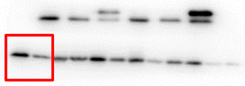

FBXO31

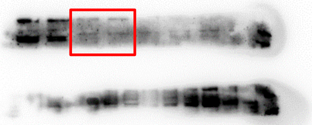

F. BGC

Actin

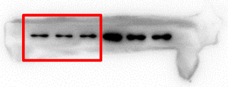

Cyclind1

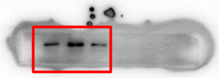

FBXO31

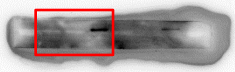

Snail

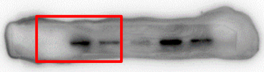

STYX

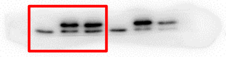

F. SGC

Actin

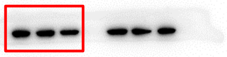

Cyclind1

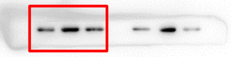

FBXO31

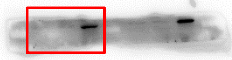

Snail

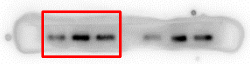

STYX

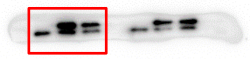

G.

IP-Myc

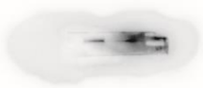

Input-Myc

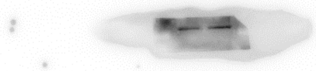

IP-STYX

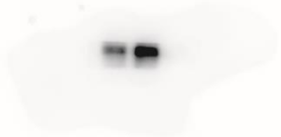

Input-STYX

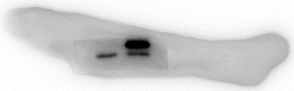

IP-Snail

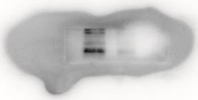

Input-Snail

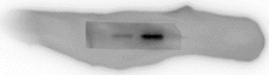

Supplement: Supplementary file 7 — Original Data File [file 41419_2022_4696_MOESM7_ESM.pdf]
